# Preliminary Studies on Endotherapy Based Application of Ozonated Water to Bobal Grapevines: Effect on Wine Quality

**DOI:** 10.3390/molecules27165155

**Published:** 2022-08-12

**Authors:** Ana Campayo, Cristina Cebrián-Tarancón, María Mercedes García-Martínez, María Rosario Salinas, Gonzalo L. Alonso, Kortes Serrano de la Hoz

**Affiliations:** 1 Cátedra de Química Agrícola, E.T.S.I. Agrónomos y Montes, Universidad de Castilla-La Mancha, Avda. de España s/n, 02071 Albacete, Spain; 2 BetterRID (Better Research, Innovation and Development, S.L.), Carretera de Las Peñas (CM-3203), Km 3.2, Campo de Prácticas-UCLM, 02071 Albacete, Spain

**Keywords:** ozone, trunk injection, spraying, colour, phenolics, volatiles

## Abstract

The application of ozonated water in the vineyard is an increasingly popular tool for disease management, but the quality of grapes and resulting wines is likely to be affected. Endotherapy, or trunk injection, is a particularly useful method to apply phytosanitary products since many fungal pathogens colonize the grapevine woody tissues. Thus, the present study aimed to evaluate the effect on wine quality of the ozonated water applied to Bobal grapevines, one of the most cultivated red varieties in Spain, through endotherapy (E) or its combination with spraying (E + S). Endotherapy was carried out four times before harvest for both E and E + S treatments, and spraying was performed 2 days before and after each endotherapy application. Grapes were harvested, vinified, and the quality of the finished wines was evaluated through several enological parameters and the phenolic and volatile composition. Both treatments resulted in less alcoholic and more acidic wines. The E treatment, although it reduced the content of phenolic acids, stilbenes and flavanols, significantly increased anthocyanins, whereas E + S decreased the overall amount of phenolics, which had different implications for wine colour. In terms of aroma, both treatments, but E to a greater extent, reduced the content of glycosylated precursors and differentially affected free volatiles, both varietal and fermentative. Thus, the dose of ozonated water, frequency and/or method of application are determining factors in the effect of these treatments on wine quality and must be carefully considered by winegrowers to establish the optimum treatment conditions so as not to impair the quality of wines.

## 1. Introduction

In a context of growing concern for more sustainable vineyard management, ozone (O_3_) in aqueous solution has recently emerged as an attractive tool to control grapevine diseases. The use of ozonated water has been proposed in integrated vineyard management to reduce epiphytic bacteria and fungi on leaves and grape bunches with efficacy similar to that of traditional chemical treatments [1], such as, for example, the active substances metrafenone, metalaxyl or copper for fungi control, whose restriction by legislation is becoming increasingly important. The fungicide properties of ozonated water have also been demonstrated in vitro and in planta against the esca-associated fungus *Phaeoacremonium aleophilum* [2]. This germicidal action is due to the strong oxidizing ability of ozone, which has higher oxidation potential than the widely used sanitizer hypochlorous acid and chlorine [3]. Additionally, the decay of ozone in aqueous solution leads to the formation of reactive oxygen species (ROS) such as the hydroxyl radical (•OH), which is an even stronger oxidant than molecular ozone [4]. As a result, ozone and derived ROS can attack numerous cellular constituents and inactivate a wide spectrum of microorganisms in a short period of time [3,5]. In this process, excess ozone auto-decomposes into oxygen due to the instability of the molecule, particularly in aqueous solution, which results in the absence of harmful residues [5].

These properties make ozone suitable for application in the food industry in both the gaseous and aqueous phases. In particular, it has been extensively used to ensure the microbiological safety and extend the shelf-life of a wide variety of food products [3,4]. In postharvest grapes, the applications of ozone are numerous: for example, the quality maintenance of table grapes during storage and retail period [6], the reduction of spoilage microorganisms in wine grapes before fermentation [7,8,9], and the replacement of sulphur dioxide in wine [10]. In blueberries it has been shown that ozone treatments induce changes in the antioxidative defence system of the fruit [11]. Grape berries accumulate secondary metabolites such as phenolic compounds [6,12,13,14,15], although they may also be reduced through alleged oxidation [7,12], or their extractability altered due to modifications in the composition, hardness and enzyme activities of skin cell walls [7,10,16,17]. Volatile organic compounds are also involved in the defensive response of grape berries to stresses, and, therefore, the aroma profile of grapes and wines is another quality parameter that undergoes changes as a result of postharvest ozone treatments [7,8,10,18,19]. As an air pollutant, the interactions of ozone with plant tissues have also been extensively studied, even in grapevines [16,17,18]. When ozone penetrates leaf tissues, the production of ROS generates oxidative stress that could eventually cause serious damage to the plant, such as the impairment of photosynthetic capacity [16,17,18,19]. To avoid ozone injury, plant cells possess detoxification mechanisms that include the action of enzymes and other antioxidants such as phenolic compounds [19,20,21,22,23]. Volatile organic compounds are also implicated in plant responses to ozone, including terpenoids for their apparent involvement in the antioxidant system and C6 compounds (such as 1-hexanol and *trans*-2-hexenal, among others), which are indicators of membrane denaturation [20,24,25]. Ozonated water has also been used to reduce fungal spoilage in postharvest blueberries [26]. In addition, when it was sprayed on grapevines as a phytosanitary treatment, an indirect impact on the quality of grapes and resulting wines —mainly related to their phenolic and aroma composition—was reported [27,28,29]. Endotherapy, also known as trunk injection, is an alternative method of applying phytosanitary products to plants. The products are directly injected into the vascular system, thus promoting their efficient use, avoiding the drift associated with spraying, and reducing the impact on the user and the environment. It is an application method primarily used in large trees and the proximity of urban areas when spraying is impractical or restricted [30]. In grapevines, endotherapy is particularly useful to control diseases in which wood pathogens are involved, as the active substance is applied directly to the infected tissues. In fact, the injection of different fungicides and chemicals into the grapevine trunk has shown promising results against the esca disease complex [31,32,33,34]. As for ozonated water, its application to grapevines exclusively through endotherapy or in combination with spraying resulted in changes in the colour and the phenolic and aroma compounds of the fruit at harvest [35]. However, no studies have been reported on the effect of ozonated water injection into the vine trunk on the quality of the resulting wine. It must be taken into account that diseases caused by fungi are responsible for the greatest losses in vineyards, representing more than 97% of the expenditure on pesticides for this crop in Spain [36].

Given that ozonated water influences the grape composition and the compounds’ extractability, we hypothesized that the quality of wines made from grapevines subjected to endotherapy-based ozonated water treatments would also be affected. Thus, the aim of this work was to evaluate the effect of ozonated water, applied to Bobal grapevines through endotherapy alone or combined with spraying, on the enological, phenolic, and aromatic quality of wines. As mentioned above, the application of ozonated water in the vineyard represents an interesting tool for disease management, but the impact of this kind of treatment on the quality of grapes and resulting wines is still unclear.

## 2. Results and Discussion

### 2.1. Effect on Enological Parameters

**Figure 1:**
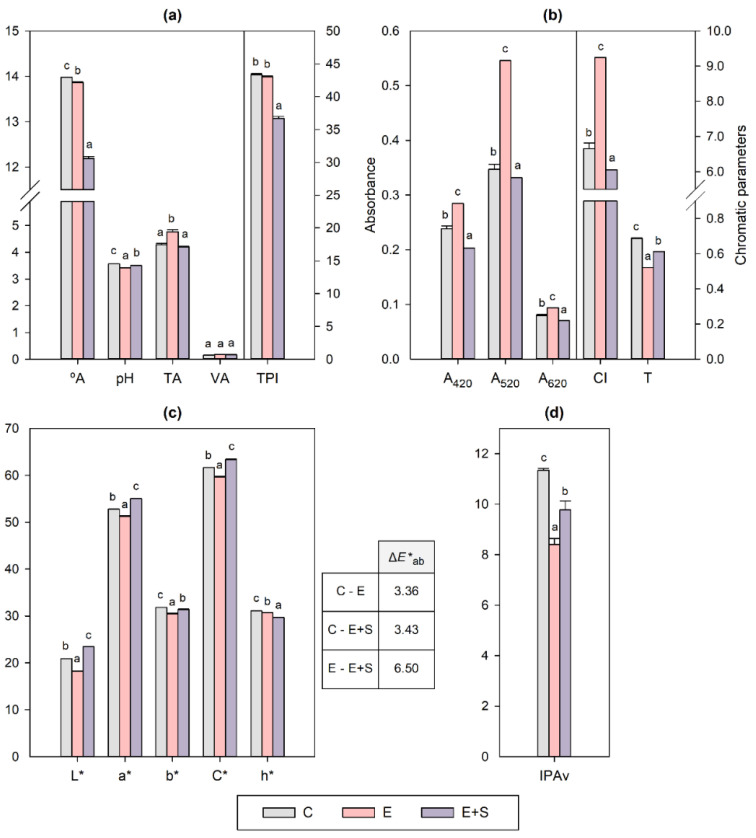
Enological parameters determined in Bobal wines, including: (**a**) classical parameters; (**b**) chromatic parameters according to Glories; (**c**) chromatic parameters according to CIELAB; (**d**) varietal aroma potential index (IPAv). C: control wine (from untreated grapevines); E: wine from grapevines treated by endotherapy; E + S: wine from grapevines treated by endotherapy and spraying; °A: Alcoholic degree (%v); TA: titratable acidity expressed as g/L of tartaric acid; VA: volatile acidity expressed as g/L of acetic acid; TPI: total phenol index; A_420_: absorbance at 420 nm; A_520_: absorbance at 520 nm; A_620_: absorbance at 620 nm; CI: colour intensity; T: tonality; L*: lightness; a*: red/green colour component; b*: yellow/blue colour component; C*: chroma; h*: hue angle; ΔE*_ab_: chromatic differences between wines. All data are expressed as mean ± SD (*n* = 2). For each parameter, different letters indicate significant differences according to the Tukey test (*p* < 0.05).

The enological parameters of the wines from control and treated grapevines, which include classical and chromatic parameters and aromatic potential, are shown in Figure 1. The alcoholic and malolactic fermentations developed properly and with similar kinetics in all of the wines (data not shown).

As regards the classical parameters (Figure 1a), both E and E + S treatments resulted in wines with lower °A than the control, especially the combined one, but still with acceptable alcohol content for red wine. The greater reduction of the °A found in E + S seems to be attributable to lower sugar content in the initial must (24.2, 24.1 and 23.4 °Brix in C, E, and E + S respectively). In grapes, reductions in carbohydrate accumulation have been described when the cumulative ozone uptake of grapevines exceeds a critical level [17]. The wines from treated grapevines were also more acidic than the control in terms of pH, particularly the E wine, which also showed a substantially higher TA. This increased acidity was already observed in the grapes from which these wines were produced [35], suggesting a delaying effect of the endotherapy-based treatments on their technological maturity. The reduction of the alcohol content in wines and the increase in their acidity are interesting strategies to counteract the effects of climate change [37]. No significant differences were found in the VA, and, although the E wine had a TPI similar to that of the control, E + S reduced this index related to the phenolic content. Adverse effects on the phenolic composition of grapes and wines have already been observed after exposure of grapevines and harvested grapes to ozone, either present in the atmosphere or applied in the aqueous or gaseous form [7,12,27], as will be further discussed.

Colour is one of the most important organoleptic features of wine. The chromatic characteristics of the studied wines were determined according to Glories and CIELAB. In the first case, the absorbances measured at 420 (yellow), 520 (red) and 620 (blue) nm and the derived parameters CI and T are shown in Figure 1b. The E treatment led to a wine with higher absorbances than the control at the three wavelengths, notably at 520 nm (+57%), which is indicative of increased intensity of yellow, blue and especially red colours. In contrast, E + S had the opposite effect, and the corresponding wine showed lower absorbances, with the greatest reduction at 420 nm (−15%). Consequently, in comparison with the control wine, the CI values were higher for E and lower for E + S. As for the tonality, both wines from treated grapevines, and particularly E, showed a higher proportion of red to yellow tones, indicated by a lower T, which is very suitable for young red wines.

In an attempt to resemble the real sensation experienced by an observer, the colour of wines was also defined by the CIELAB coordinates (L*, a* and b*) and derived magnitudes (C* and h*), which are shown in Figure 1c. In comparison with the control, the E wine was darker, as indicated by the lower value of L*, but with slightly lower red (a*) and yellow (b*) components, and, therefore, lower chroma (C*). This wine also showed a lower hue angle (h*) than the control because the yellow component decreased barely more than the red one. In contrast, the E + S wine showed greater clarity (L*) but higher C* since, although b* slightly decreased, a* significantly increased in comparison with the control wine, which also resulted in a lower h*. Except for L* and partially h*, the other CIELAB parameters showed no apparent correlation with CI, T and the absorbances from which they derive (Figure 1b). Accordingly, some authors have only found a straightforward relationship between CI and L* [38]. The reason for these differences is that the Glories parameters, commonly used in wineries, are only based on three wavelengths, while the CIELAB ones are calculated from a much wider range of absorbances within the visible spectrum. The overall colour difference between the wines from treated grapevines and the control, calculated as ΔE*ab, was similar for both treatments (Figure 1c). Since ΔE*ab of around 3.0 units is estimated as an acceptable tolerance level by the human eye for red wines [39], both E and E + S wines would be visually distinguishable from the control. Nevertheless, the colour difference was even greater between the two wines from treated grapevines, with ΔE*ab being nearly the double of the respective variations with the control. Both Glories and CIELAB parameters indicate that the dose of aqueous ozone (i.e., volume) and/or the application frequency, or even the application method itself, are determining factors in the impact of these treatments on wine colour.

The wine aroma potential concerning the content of glycosylated aroma precursors was evaluated using the varietal aroma potential index (IPAv, Figure 1d). In wines, this index is an estimation of the aroma compounds extracted from grapes that remain glycosylated and, therefore, in a non-volatile form, but could potentially be hydrolysed and contribute positively to wine aroma. The aglycones mainly include alcohols, terpenols, phenols and C13-norisoprenoids [40]. Regarding the effect of ozonated water, the wines from treated grapevines presented lower IPAv values than the control, especially that of the E treatment. This can mean two things: more glycosylated precursors were released during winemaking, or lower amounts were extracted from the starting grapes, either because these had a lower content or a worse extractability. The analysis of the starting grapes revealed lower amounts of these precursors in the treated ones [28]. However, contrary to the behaviour observed in the grapes, the greatest IPAv drop was found in the E wine, indicating that despite decreasing the content of these compounds in the starting material, the E treatment could promote a higher release of aglycones and/or a lower extraction of glycosylated precursors than C and E + S during winemaking. The higher hydrolysis could be partly favoured by the higher acidity found in E-treated grapes [41] and wine (Figure 1a). The negative effect of ozone on the glycosylated aroma precursors of wines, in the sense that they contain fewer potential odorants, has already been reported when sprayed in the aqueous form on Bobal grapevines [28]. The impact of postharvest ozone treatments on the bound aroma compounds of wines has not been studied, but in grapes they have been seen to affect the glycosylated fraction differently depending on factors such as the dose of ozone, the exposure time, the weight loss of withered grapes, the grape variety and the compound considered [9,13,42]

### 2.2. Effect on Phenolic Compounds

The detailed phenolic composition of the wines is shown in Table 1. The low-molecular-weight phenolic compounds identified have been classified into phenolic acids, stilbenes, flavanols, flavonols and anthocyanins.

The E treatment resulted in a wine with the highest total phenolic content due to the significant increase in anthocyanins (+33%), although the content of other families such as phenolic acids (–36%), stilbenes (–19%) and flavanols (–22%) was reduced in comparison with the control. It is well known that certain phenolic compounds contribute to wine colour, with anthocyanins being responsible for red, purple and blue pigmentation of red grapes and wines. Therefore, the increased anthocyanin content in the E wine, although not reflected in the TPI, is consistent with its higher absorbance at 520 and 620 nm (Figure 1b), as well as with the effect observed in the respective grapes [41]. The decrease in the total concentration of phenolic acids, stilbenes and flavanols could explain the lower a* value observed in the E wine (Figure 1c) because, despite being non-coloured, some of them have been described as copigments of anthocyanins [43]. Furthermore, the lowest total phenolic content was detected in the wine from the more intensive E + S treatment, which decreased the overall concentration of phenolic acids (–27%), stilbenes (–18%), flavanols (–18%), flavonols (–22%), and anthocyanins (–14%) compared to their levels in the control wine. This agrees with the lower TPI and CI, and the higher L* observed in the E + S wine (Figure 1a–c), along with the results concerning the phenolic content, anthocyanin extractability and chromatic characteristics of the starting grapes [41]. In addition to the phenolic composition, the pH of the wines (Figure 1a) could contribute to the colour differences detected, as stated by Heras-Roger et al. (2016) [43].

It is well known that the accumulation of phenolic compounds is one of the strategies of plants to avoid and tolerate ozone stress, but their production implies a high metabolic cost and is achieved at the expense of other compounds or even plant growth [19,21,22,23].

Depending on the genotype and sensitivity to ozone, the plant invests in efficient high-cost or less efficient low-cost antioxidants [19]. However, the exposure to elevated ozone may result in reduced photosynthesis and, therefore, lower carbon availability to synthesize phenolics and other antioxidants [19]. In the specific case of grapevines, Merlot grapes from plants exposed to ozone-rich air were characterized by lower yields and polyphenol content than the ones enclosed in open top chambers with filtered air [16]. When it comes to ozonated water, spraying treatments have shown a positive, negative or no effect on the phenolic content of Vermentino and Bobal grapes or wines [27,28,29]. These differences could be attributed to the cultivar, the dose, the frequency of application, and the environmental conditions and consequent influence on the ozone uptake by the plants.

In the case of phenolic acids, both ozonated water treatments reduced the content of gallic, vanillic, *trans*-caffeic and *trans*-caftaric acids compared to their levels in the control wine, while syringic and *trans*-p-coutaric acids were not significantly modified. This decrease was more pronounced in wine E for gallic acid (–45%) and E + S for *trans*-caffeic acid (–38%). The grapes subjected to treatment E presented a lower potential contribution of seed tannins [35], which, in addition to the contact with oak wood, are one of the main sources of gallic acid in wine [44]. In white wine grapes, Carbone and Mencarelli (2015) [12] also reported that short-term postharvest ozone treatments caused a large decrease in phenolic acids, which the authors attributed to the oxidative role of ozone and the low redox potential of these compounds compared to other phenolics [12].

Regarding the stilbenes, the contents of both *trans*-resveratrol and its glucoside piceid were also negatively affected by the treatments, resulting in wines with approximately 18% less stilbene content. Stilbenes are naturally present in grapes, mainly in their skin, but are also produced in response to fungal attacks and other stresses [44]. Given the beneficial health effects attributed to *trans*-resveratrol, stress-induced stilbene synthesis by external stimuli such as ozone has been exploited in postharvest grapes [45]. However, an elicitor effect has not always been observed. For example, ozone fumigation on two wine grape cultivars and ozonated water treatments on harvested grapes did not induce stilbene production in leaves and grapes, respectively [14,18]. Likewise, Río Segade, Vincenzi et al. (2019) [15] found that short-term postharvest ozone treatments enhanced the accumulation of stilbenes in subsequently dehydrated wine grapes, while long-term and continuous treatments did not activate their production.

Flavanols followed the same trend as stilbenes, with a 20% average reduction of (+)-catechin and (–)-epicatechin in the wines from treated grapevines. The use of ozone at postharvest has been seen to favour the accumulation of flavanols in table grapes [6], but also to reduce their content in wine grapes when compared to air-exposed ones [12]. Furthermore, their extraction during skin maceration has proven to be affected by postharvest ozone exposure [15,46]. In general, analogous treatments, but performed exclusively through spraying on the same cultivar, did not alter or even increased the content of phenolic acids, stilbenes and flavanols in wines [28]. This suggests that the use of endotherapy as the delivery method could be responsible for the adverse impact observed on these phenolic compounds, either because their oxidation was greater than their production, their extractability was impaired or their synthesis was inhibited, probably at the expense of other compounds such as anthocyanins. Additionally, the decrease in phenolic acids, stilbenes and flavanols was similar in both treatments and, in general, showed no apparent correlation with their intensity.

In the case of flavonols, the combination of endotherapy and spraying clearly caused a reduction in the content of all those identified in the wine, except the glucuronide and glucoside of myricetin, where the decrease compared with the control was not statistically significant. Although to a lesser extent, the E treatment also reduced the content of quercetin aglycone and the sum of its glucuronide and glucoside. However, this less intensive treatment did not alter the amount of myricetin and its galactoside, kaempferol and its glucoside, and quercetin 3-O-galactoside, and even favoured the glucuronide and glucoside of myricetin and the glucoside or galactoside of laricitrin. The reduction of quercetin glycosides and aglycone even by the milder treatment E could be ascribed to their high antioxidant capacity and consequently to an allegedly increased susceptibility to be oxidized. Indeed, quercetin exhibits higher antioxidant activity than myricetin and kaempferol, and is also considered one of the phenolic compounds with the highest antioxidant power [47]. Quercetin-type flavonols were also the most affected by the combined treatment. Previous studies have reported that spray treatments of ozonated water did not adversely affect or even enhanced the flavonol content of Bobal wines [28]. These treatments were, along with E, less intensive than E + S, which suggests a greater susceptibility of flavonols to increasing doses of ozonated water. In this line, it has been reported that the content of polyphenols remained unaffected by an ozone shock treatment before dehydration, whereas it was considerably decreased after a longer treatment, presumably due to oxidation [7]. Since flavonols are localized together with anthocyanins in skin vacuoles and follow similar extraction patterns [48], the lower anthocyanin extractability found in E + S grapes [41] could also affect flavonols and be one of the causes of their lower content in the corresponding wine.

As for anthocyanins, the ozonated water treatments showed an opposite trend. In all of the wines, the non-acylated delphinidin, cyanidin, petunidin, peonidin and malvidin monoglucosides, the acetylated peonidin and malvidin, and the p-coumaroylated petunidin and malvidin were quantified. All of the non-acylated and acylated anthocyanins were enhanced in wine when the grapevines had been treated through endotherapy, while a significant reduction was observed when additional spraying applications were performed. In this case, the analysis of the E + S grapes revealed that the accumulation of anthocyanins was enhanced, but their extractability from the berry skin was hindered [41]. The same pattern was observed for spraying treatments of different intensity carried out on Bobal grapevines [27,28], indicating that above a certain dose of pulverized ozonated water, the increased anthocyanin accumulation is offset by a lower extractability. Differences in the extraction of anthocyanins can be linked to changes in the skin cell wall composition and mechanical properties, which have been described after fruit exposure to ozone [7,15]. However, the effect of ozone on the extractability of anthocyanins is dependent on the variety and the exposure time [15,46]. The boosting effect of treatment E also affected vitisin B, which showed a higher concentration in the resulting wine than in the control. This pyranoanthocyanin is formed mainly during fermentation by the reaction of malvidin 3-O-glucoside with the yeast by-product acetaldehyde and is characterized by a reddish-orange colour [49]. Therefore, the 63% increase in the content of this pigment compared to the control wine could partly explain the higher A420 observed in the E wine (Figure 1b).

In addition to the influence of the dose, frequency and method of application, the response of phenolic compounds to ozonated water treatments could be related to their location in the grape berry. Anthocyanins and flavonols are located exclusively in the berry skin, while phenolic acids, stilbenes and flavanols are also present in the seeds or even in the flesh [50]. This would explain why the treatment E, in which the ozonated water did not come into direct contact with the grape surface, was less detrimental to flavonols and even favoured the anthocyanin content in wine, while when this treatment was supplemented with spraying applications, the negative effect on these compounds became noticeable. In addition, the period in which phenolic compounds are synthesized may play a role in their response to the treatments. The synthesis of phenolic acids, stilbenes, flavanols and flavonols begins before veraison, and in many cases during the early stages of fruit development, while the accumulation of anthocyanins starts from veraison and reaches its maximum in the latest phases of maturation [50]. This means that anthocyanins are less exposed to the oxidation triggered by ozone, especially in milder treatments that involve a lower dose from the onset of ripening. In this work, E and E + S treatments, and previously S1 and S2 [27,28], were shown to stimulate the synthesis of anthocyanins in grapes, although a lower extractability caused by the treatments E + S and S2 resulted in wines with lower concentrations of these compounds. An increase in the anthocyanin content of grapes is of general interest in the wine industry, but the extractability of these compounds must be considered. Therefore, the winemaking technique must be adjusted to the characteristics of the raw material according to the wine to be produced.

### 2.3. Effect on Volatile Compounds

The free volatile compounds quantified in the wines are shown in Table 2. They have been classified into six families: acids, alcohols, acetates, ethyl esters, terpenoids, and volatile phenols. The concentrations of the volatile compounds identified in control and treated wines were similar to those described for Bobal wines from the same plot [28] and La Mancha region [51]. The aroma profile of the wines was modified by the ozonated water treatments, among which E showed a higher influence. In general, the E treatment led to increased total contents of acids and acetates, while both treatments decreased the total amount of alcohols compared to the control.

There was a clear effect of the E treatment concerning acids since significantly higher concentrations of decanoic, hexanoic and octanoic acids were detected in the resulting wine, increasing by 81% the total amount of volatile acids compared to the control. In the case of E + S, although the corresponding wine presented average concentrations of these acids higher than in the control, the differences were not statistically significant. Both hexanoic and octanoic acids had an OAV higher than 1 in all of the wines (Table 2), so they can be expected to contribute significantly to wine aroma, especially in the case of E. Although their odour is described as fatty, rancid or cheesy, these volatiles are considered to contribute positively to the complexity and equilibrium of wine aroma [44]. Most of the fatty acids found in wine are formed from acetyl-CoA via yeast lipid metabolism. The major source of acetyl-CoA for fatty acid synthesis during alcoholic fermentation is acetic acid, formed by oxidation of acetaldehyde [44]. Therefore, the increased presence of volatile fatty acids in the E wine could be related to a higher concentration of acetaldehyde, which would also be consistent with the higher content of vitisin B (Table 1). In the literature, no elicitor effect of ozone on the production of acetaldehyde by plants or postharvest fruits has been described. In wine, this aldehyde is formed by yeasts mainly from sugar, but also amino acids, and during the oxidation of ethanol to acetic acid, either microbiologically or under oxidative conditions [57]. However, all of the wines were elaborated under the same conditions (yeast strain, temperature, aeration, SO_2_ addition), and the treated wines showed low and similar VA values compared to the control wine (Figure 1a). It is also excluded that the oxidative conditions were directly generated by the ozone uptake by the plants due to the time elapsed between the last applications and harvest. The fatty acid composition in the grape must could also influence the formation of yeast-derived volatile compounds by regulating the synthesis of acetyl-CoA [58]. The addition of low concentrations of unsaturated fatty acids such as linoleic acid or oleic acid in the must has been demonstrated to facilitate the production of medium-chain fatty acids and acetate esters, especially isoamyl acetate, during red wine fermentation [58]. The accumulation of ROS after ozone uptake leads to the formation of unsaturated fatty acids [24], activating some genes involved in their synthesis [42]. Eventually, these fatty acids could be oxidized to produce C6 compounds, some of which tended to increase in grape must after the E and E + S treatments, particularly in the former [41]. Regarding alcohols, both treatments resulted in wines with lower amounts than in the control, mainly due to the decrease in amyl alcohols, i.e., 2-methyl-1-butanol and 3-methyl-1-butanol. These alcohols with fusel-like odour, in addition to being the most abundant, exceeded their odour threshold in all of the wines (Table 2). Benzyl alcohol was also reduced in both treated wines, but other alcohols such as 1-hexanol and 2-phenylethanol showed concentrations similar to those in the control wine. Benzyl alcohol can proceed from the fermentation process but primarily from grapes, where it can be found in the glycosylated form. Therefore, the lower levels of benzyl alcohol could be related to the IPAv reduction detected in the treated grapes [41]. This benzenoid, like the non-volatile phenolic compounds, is formed from phenylalanine through the phenylpropanoid pathway [59], so its synthesis in grapes may have been impaired to preferentially form anthocyanins or other antioxidants. Contrarily, amyl alcohols are produced by yeasts from sugars and amino acids [44], so their reduced content in the wines from treated grapes could be the result of lower availability of these substrates in the grape must. However, their reduction was probably due to the increased formation of the corresponding acetate esters, as indicated by the significantly higher concentration of isoamyl acetate, the ester formed from 3-methyl-1-butanol and acetic acid (Table 2). In addition to alterations in the precursors of these volatiles or a possible transformation into other compounds, changes in the fermentative aroma could also be related to alterations in grape microbiota. Postharvest ozone treatments on wine grapes have been proven to modify the yeast population present on berry surfaces and during inoculated fermentations, and consequently the chemical composition of wines [8]. Two main classes of esters are present in wine and contribute to its fruity and floral aroma: acetate esters and ethyl esters, most of which are formed during fermentation and storage [44]. As for the impact of ozonated water, the E treatment caused an increase in the content of acetate esters in the resulting wine (Table 2). In particular, the concentrations of ethyl acetate, hexyl acetate, isoamyl acetate and 2-phenylethyl acetate were higher in the E wine than in the control. The acetate ester of ethanol, whose odour is described as fruity and solvent-like, was the most abundant and presented an OAV above the unit in all the wines, as was the case with isoamyl acetate, whose aroma descriptor is banana. The combined treatment also favoured the presence of the acetate ester of 1-hexanol and isoamyl alcohol, while none of the treatments altered the content of linalyl acetate. The higher concentration of certain acetate esters in the treated wines must be related to the activity of acetyltransferase enzymes or the availability of the two substrates: acetyl-CoA and respective alcohols [44], which are either products of sugar and amino acid yeast metabolism (ethanol, isoamyl alcohol and 2-phenylethanol) or grape-derived (1-hexanol). Again, increased availability of acetyl-CoA in the medium of the treated musts/wines is suggested, which would lead to the increased synthesis of volatile fatty acids and acetate esters (Table 2).

The other major class of wine esters comprises those formed between ethanol and organic acids, called ethyl esters (Table 2). In comparison with the control, both wines made from treated grapevines showed lower contents of ethyl lactate. This ester, associated with malolactic fermentation, was notable for its abundance but did not exceed its odour threshold in any of the wines. The E + S treatment did not modify the content of any other ethyl ester, but E significantly increased the amounts of diethyl succinate, ethyl butyrate, ethyl decanoate, ethyl hexanoate and ethyl octanoate. The increased concentrations of the last three ethyl esters coincide with the higher content found in this wine for the fatty acids from which they derive, i.e., decanoic, hexanoic and octanoic acids (Table 2). Concerning their contribution to wine aroma, the ethyl esters of butyric, hexanoic and octanoic acids, which are associated with fruity odours, showed concentrations above their odour thresholds in all of the wines, although a greater contribution could be expected in E. Similarly, the sensory evaluation of Petit Verdot wines made from ozone-treated grapes revealed a strong fruity aroma that the authors attributed to a supposed lower loss of esters due to the protective effect of phenolic compounds [10]. With respect to ethyl dihydrocinnamate and ethyl vanillate, both volatiles had similar concentrations in control and treated wines.

The terpenoid family includes terpenes and C13-norisoprenoids, which impart desirable floral and fruity aromas. Terpenoids in wine mainly have their origin in grapes, where they are commonly found as non-volatile glycosides. The control and treated wines generally showed similar concentrations of these compounds, although some individual differences were found (Table 2). For example, in comparison with the C wine, the amount of citronellol was reduced in E, while geranyl acetone was increased. These terpenoids were not modified in the E + S wine, but both treatments enhanced the linalool content by approximately 37%. None of the ozonated water treatments significantly altered the wine composition in relation to farnesol, geraniol, β-ionone, nerol and nerolidol. An increased content of certain free terpenoids was already detected in the corresponding starting grapes [41], as well as in grapes and wines from grapevines subjected to ozonated water spraying [27,29], even though this kind of treatment generally reduced the content of glycosylated precursors [27,41]. Terpenoids are synthesized in plants and postharvest fruits for their protective role against oxidative stress caused by ozone [25,42]. Additionally, a greater release of aglycones from the glycosylated precursors during winemaking may have occurred, as has been previously suggested for the E wine. Although terpenoids originate primarily from grapes, the different effects observed among compounds or between grapes and wines may be due to the biotransformation capacity of yeasts [60]. The content of volatile phenols in wines was not affected by the ozonated water treatments. This fraction of the wine aroma can arise from grape glycosides and yeast metabolism of phenolic compounds [44]. In particular, eugenol and guaiacol were detected, the latter showing an OAV higher than 1 in all of the wines. Accordingly, these volatiles remained unchanged in wine when ozonated water was sprayed on grapevines [28]. All of these results concerning volatile compounds demonstrate that the endotherapy-based ozonated water treatments E and E + S not only affected the varietal aroma of wines, but also those formed in the fermentation process, as was already observed for spraying treatments [28]. In addition, the different effect observed for each treatment proves that the dose, frequency and/or method of application influences the impact of ozonated water on the volatile composition of wines.

## 3. Materials and Methods

### 3.1. Grapevines

The experiments were carried out during the year 2017 in an unirrigated plot of *Vitis vinifera* L. cv. Bobal grapevines, as described by Campayo et al. (2020) [41]. Briefly, the plot was in Casas de Haro (Cuenca, Castilla-La Mancha, Spain) at an altitude of 730 m, latitude 39°18′ N and longitude 2°13′ W, and contained 18-year-old grapevines grown on a vertical trellis system and 110-Richter rootstock.

### 3.2. Ozonated Water

Ozonated water was generated on site immediately before the treatments by a specific prototype designed for agriculture (Nutricontrol, S.L., Cartagena, Spain). The equipment consisted of an ozone generator from atmospheric oxygen and a 500 L thermally insulated tank, which was filled with water from a well located in the vineyard with an approximate temperature of 15 °C. The prototype contained a sensor to measure in millivolts (mV) the oxidation–reduction potential (ORP) of the water and a controller to automatically maintain the desired ORP, which was 875 ± 25 mV throughout the treatments.

### 3.3. Grapevine Treatments

Two different ozonated water treatments were applied to the grapevines using different application strategies:

- Endotherapy (E): direct injection of ozonated water into the trunk through a hole (3 mm diameter, 1 cm depth) drilled above the graft union and sealed with a hermetic cannula connected to the ozonated water tank. The volume of ozonated water injected in each grapevine and application, under a maximum pressure of 1 bar, was approximately 0.5% of the trunk volume in order to be distributed through the xylem and reach the different parts of the plant. All of these parameters were selected based on preliminary tests (data not shown). Ozonated water was applied four times before harvest, in particular before flowering, after the fruit set, at veraison and during the ripening period.
- Combination of endotherapy and foliar spraying (E + S): the ozonated water was applied through endotherapy in the same manner and on the same dates as in the E treatment, while spraying was performed 2 days before and after each endotherapy application. Approximately 300 mL of ozonated water were sprayed per plant and application to cover the entire canopy.

Each treatment was performed on 15 randomly selected plants, and 15 untreated plants were used as controls (C), as described by Campayo et al. (2020) [41]. A buffer row between the control and each treatment was left to avoid the drift effect. The plants under study were visually healthy to minimize the effect of pests or diseases on the quality of grapes and resulting wines. They were located in a homogeneous portion of the plot and the outer rows were avoided. The treatments were carried out early in the morning, when the environmental temperature was around 20 °C. Grapes were manually collected when the optimal technological maturity of the control ones was achieved (most suitable °Baumé/titratable acidity ratio).

### 3.4. Winemaking

Grapes from control (C) and each treatment (E and E + S) were vinified separately and in duplicate. A traditional red winemaking method was followed, where approximately 4 kg of grapes of each condition (C, E and E + S) were manually destemmed and crushed. Potassium metabisulfite (20 mg/kg) was added to the resulting grape mass, which was placed in 7 L methacrylate tubes for maceration and fermentation. Grape skins were kept submerged in must with the aid of a plunger, and all of the tubes were placed in a multitube fermenter (Martínez Solé y Cía, S.A., Villarrobledo, Spain). To carry out the alcoholic fermentation, which took place at 24 ± 1 °C, Lalvin EC1118 active dry yeast (25 g/hL) supplemented with Go-Ferm Protect Evolution was inoculated, and in subsequent days Nutrient Vit Nature and Nutrient Vit Blanc were added according to instructions of the supplier (Lallemand, Spain). °Brix and temperature were measured, and grape skins were plunged daily. When °Brix was constant at approximately 7, alcoholic fermentation was considered finished, and the wines were pressed manually to remove skins and seeds. Malolactic fermentation, which was carried out at 18 ± 1 °C, was induced by adding 1.5 g/hL of commercial bacteria (Lalvin VP41, Lallemand, Spain) after lees removal. It was monitored by daily measurement of the pH and the concentrations of malic and lactic acids by HPLC-RID according to Martínez-Gil et al. (2013) [61]. Malolactic fermentation was considered finished when the concentration of malic acid was constant and below 0.1 g/L. Then, SO^2^ concentration was corrected by adding 80 mg/L of potassium metabisulfite, and wines were bottled and frozen at −18 °C until further analysis.

### 3.5. Analytical Methods

#### 3.5.1. Wine Enological Parameters

The classical parameters of wines, such as the alcoholic degree (°A), pH, titratable acidity (TA, g/L of tartaric acid) and volatile acidity (VA, g/L of acetic acid), were analysed by Fourier transform-infrared spectroscopy equipment (FT-IR Bacchus 3 MultiSpec, TDI, Barcelona, Spain) using the methods established by the International Organisation of Vine and Wine [62] as a reference.

The total phenol index (TPI) of wines was obtained by measuring the absorbance at 280 nm [63]. The chromatic parameters analysed were colour intensity (CI), tonality (T) and CIELAB coordinates. To determine CI and T, the absorbances at 420, 520 and 620 nm were measured, and then CI was calculated as the sum of these absorbances (A420 + A520 + A620) and T as the ratio A420/A520 [64]. CIELAB coordinates were determined according to the regulations established by the Commission Internationale de l’Eclairage [65], by measuring the transmittance from 380 to 780 nm at 5 nm intervals followed by a calculation using the software Color of Wines-2001 (Perkin-Elmer Hispania, Madrid, Spain). Overall colorimetric differences between wines were calculated using the expression ΔE*ab = (Δa*2 + Δb*2 + ΔL*2)1/2 [66]. Spectrophotometric determinations were made by Lambda 25 UV–Vis equipment (Perkin Elmer, Norwalk, CT, USA), using 1 and 0.1 cm path length cells for TPI and chromatic parameters, respectively, but the absorbances were referred to 1 cm to calculate the latter. Before measuring the chromatic parameters, wines were filtered through a PVDF Durapore filter of 0.45 μm (Millipore, Bedford, MA, USA).

The varietal aroma potential index (IPAv) of wines was analysed using a commercially available kit (Teknokroma S.A., Barcelona, Spain). The method was based on the one described by Salinas et al. (2012) [40], but modified to enable spectrophotometric determination of the glycosyl glucose released from the glycosylated aroma precursors by acid hydrolysis. All analyses were conducted in duplicate on each wine replicate (*n* = 2).

#### 3.5.2. Determination of Low Molecular Weight Phenolic Compounds by HPLC-DAD

The detailed phenolic composition of wines was analysed according to Pardo-García et al. (2014) [67]. Before the analysis, wines were filtered through a PVDF Durapore filter of 0.22 μm (Millipore, Bedford, MA, USA) and a volume of 20 µL was injected into an Agilent 1200 high performance liquid chromatograph (HPLC, Palo Alto, CA, USA) equipped with a Diode Array Detector (DAD, Agilent G1315D, Palo Alto, CA, USA) coupled to an Agilent ChemStation (version B.03.01) data-processing station. Separation was performed on a reversed-phase Zorbax-Eclipse XDB-C18 (4.6 mm × 150 mm, 5 μm particle sizes) and a precolumn of the same material at 30 °C. The HPLC grade solvents used were water/formic acid/acetonitrile (97.5:1.5:1 *v*/*v*/*v*) as solvent A and acetonitrile/formic acid/solvent A (78.5:1.5:20 *v*/*v*/*v*) as solvent B. The elution gradient for solvent B was as follows: 0 min, 5%; 2 min, 10%; 7 min, 14.5%; 10 min, 18.5%; 12 min, 20%; 17 min, 20%; 28 min, 30%; 30 min, 30%; 32 min, 50.5%; 38 min, 80%; 40 min, 100%.

The standards employed to identify and quantify the phenolic compounds were gallic acid, *trans*-caffeic acid, vanillic acid, syringic acid, *trans*-p-coumaric acid, *trans*-resveratrol, piceid-*trans*-resveratrol, (+)-catechin, (–)-epicatechin, quercetin, and malvidin 3-O-glucoside (Sigma-Aldrich, Steinheim, Germany). The identification was carried out by comparison with their corresponding UV-Vis spectra and retention time of their pure standards. The identification of compounds for which no standard was available was carried out as previously described by Pardo-García et al. (2014) [67], using their respective absorption maxima in the UV-Vis region and retention times as additional means of identification. Quantification was based on calibration curves of the respective standards at five different concentrations (R^2^ > 0.98). Acids *trans*-caftaric and *trans*-p-coutaric were tentatively identified and quantified as *trans*-caffeic acid and *trans*-p-coumaric acid, respectively. Flavonols and anthocyanins were tentatively identified and quantified as quercetin and malvidin 3-O-glucoside equivalents, respectively. All analyses were conducted in duplicate on each wine replicate (*n* = 2).

#### 3.5.3. Determination of Volatile Compounds by SBSE-GC-MS

Wine volatile compounds were determined according to the method described by Campayo et al. (2020) [28]. Their extraction was carried out by stir-bar sorptive extraction (SBSE). Later analysis was performed using an automated thermal desorption unit (TDU, Gerstel, Mülheim and der Ruhr, Germany) mounted on an Agilent 7890A gas chromatograph system (GC) coupled to a quadrupole Agilent 5975C electron ionization mass spectrometric detector (MS, Agilent Technologies, Palo Alto, CA, USA). The GC system was equipped with a fused silica capillary column (BP21 stationary phase, 30 m length, 0.25 mm I.D. and 0.25 μm film thickness) (SGE, Ringwood, Australia), and the carrier gas was helium with a constant column pressure of 20.75 psi. The GC oven temperature was programmed to 40 °C (held for 2 min), raised to 80 °C (5 °C/min, held for 2 min), raised to 130 °C (10 °C/min, held for 5 min), raised to 150 °C (5 °C/min, held for 5 min), and then raised to 230 °C (10 °C/min, held for 5 min). The MS was operated with an ionization energy of 70 eV. The temperature of the MS transfer line was maintained at 230 °C. MS data acquisition was carried out in positive scan mode. The MS identification and quantification were performed in the single ion-monitoring mode using the characteristic *m*/*z* values of the volatiles with the aid of the NIST library and confirmed with the mass spectra and retention time of their pure standards (Sigma-Aldrich, Steinheim, Germany). 3-Methyl-1-pentanol was used as internal standard. Quantification was based on calibration curves of the respective standards at five different concentrations (R^2^ = 0.95–0.99). 2-Methyl-1-butanol was quantified together with 3-methyl-1-butanol with the calibration curve of the latter. All analyses were conducted in duplicate on each wine replicate (*n* = 2). The specific contribution of each volatile compound to the overall wine aroma was determined by calculating the odour activity value (OAV) as the ratio between the concentration of the compound and its odour threshold [54].

### 3.6. Statistical Analysis

The statistical analysis of the data was performed using SPSS statistics software package version 23.0 for Windows (SPSS, Chicago, IL, USA). Data were processed using the one-way analysis of variance (ANOVA). Differences between means were compared using the Tukey post hoc test at 95% confidence interval (α = 0.05).

## 4. Conclusions

Endotherapy-based ozonated water treatments, whose purpose is the management of grapevine diseases, induced a defence response in grape berries that consequently modified the enological, phenolic, and aromatic quality of the wines. Both E and E + S treatments resulted in less alcoholic and more acidic wines, probably due to a delaying effect on the technological maturity of grapes, while their VA was unaffected. In terms of wine phenolic composition, the E treatment decreased the content of phenolic acids, stilbenes and flavanols, but not the total phenolic content due to a significant increase in anthocyanins. However, E + S decreased the overall amount of phenolics, including phenolic acids, stilbenes, flavanols, flavonols, and anthocyanins. Consequently, differences estimated as visually perceptible were found in the chromatic characteristics of the wines, including increased darkness in E, increased clarity in E + S, and a higher proportion of red to yellow colours in both. Regarding wine aroma, both ozonated water treatments decreased the content of glycosylated precursors, probably due to a previous reduction in the starting grapes. The greater IPAv reduction, along with the increased content of certain free terpenoids in the E wine, suggests that this treatment could also lead to a higher release of aglycones during winemaking. The ozonated water treatments not only affected the varietal aroma of the wines, but also those formed in the fermentation process. In this regard, the E treatment led to increased contents of acids, acetate esters and certain ethyl esters, but reduced contents of alcohols and ethyl lactate. Whereas the combined treatment also enhanced the content of certain acetates and decreased alcohols and ethyl lactate, it generally had less impact on the aroma profile of the wine. All of these findings demonstrate that the dose of ozonated water and the frequency and/or method of application are determining factors in the effect of these treatments on wine quality. Thus, preliminary tests are recommended to determine —for each variety and plot— the optimal treatment conditions for effective disease control without altering, or even improving, the quality of grapes and wines.

## Figures and Tables

**Table 1:** Phenolic compounds determined in Bobal wines.

Treatments	λ (nm) ^a^	C	E	E + S
**Phenolic acids (mg/L)**				
Gallic acid	280	25.14 ± 0.27 ^c^	13.72 ± 0.25 ^a^	17.34 ± 0.12 ^b^
*trans*-Caffeic acid	324	0.24 ± 0.01 ^c^	0.19 ± 0.00 ^b^	0.15 ± 0.01 ^a^
Vanillic acid	256	3.33 ± 0.14 ^b^	2.47 ± 0.10 ^a^	2.33 ± 0.15 ^a^
Syringic acid	280	2.54 ± 0.07 ^a^	2.44 ± 0.06 ^a^	2.24 ± 0.16 ^a^
*trans*-Caftaric acid	324	3.34 ± 0.06 ^b^	3.11 ± 0.03 ^a^	3.05 ± 0.02 ^a^
*trans*-*p*-Coutaric acid	308	0.87 ± 0.05 ^a^	0.88 ± 0.04 ^a^	0.78 ± 0.07 ^a^
Σ Phenolic acids		35.46 ± 0.45 ^c^	22.82 ± 0.30 ^a^	25.89 ± 0.53 ^b^
**Stilbenes (mg/L)**				
*trans*-Resveratrol	308	0.86 ± 0.03 ^b^	0.72 ± 0.01 ^a^	0.71 ± 0.01 ^a^
Piceid-*trans*-resveratrol	308	0.88 ± 0.01 ^b^	0.69 ± 0.01 ^a^	0.72 ± 0.02 ^a^
Σ Stilbenes		1.74 ± 0.01 ^b^	1.41 ± 0.00 ^a^	1.43 ± 0.00 ^a^
**Flavanols (mg/L)**				
(+)-Catechin	280	24.31 ± 0.38 ^b^	19.30 ± 0.35 ^a^	19.69 ± 0.14 ^a^
(–)-Epicatechin	280	17.72 ± 0.11 ^c^	13.36 ± 0.04 ^a^	14.75 ± 0.03 ^b^
Σ Flavanols		42.03 ± 0.48 ^c^	32.66 ± 0.30 ^a^	34.44 ± 0.11 ^b^
**Flavonols (mg/L)**				
Myricetin 3-O-galactoside	365	0.29 ± 0.00 ^b^	0.26 ± 0.00 ^ab^	0.23 ± 0.02 ^a^
Myricetin 3-O-glucuronide+glucoside ^b^	365	4.89 ± 0.11 ^a^	6.09 ± 0.14 ^b^	4.41 ± 0.12 ^a^
Quercetin 3-O-galactoside	365	0.63 ± 0.02 ^b^	0.54 ± 0.02 ^b^	0.42 ± 0.03 ^a^
Quercetin 3-O-glucuronide+glucoside ^b^	365	7.22 ± 0.01 ^c^	6.38 ± 0.02 ^b^	5.20 ± 0.03 ^a^
Laricitrin 3-O-glucoside/galactoside ^c^	365	1.01 ± 0.00 ^b^	1.14 ± 0.01 ^c^	0.94 ± 0.02 ^a^
Kaempferol 3-O-glucoside	365	0.49 ± 0.01 ^b^	0.43 ± 0.01 ^b^	0.37 ± 0.02 ^a^
Syringetin 3-O-glucoside	365	2.14 ± 0.03 ^b^	2.15 ± 0.01 ^b^	1.74 ± 0.04 ^a^
Myricetin	365	0.37 ± 0.00 ^b^	0.37 ± 0.00 ^b^	0.30 ± 0.01 ^a^
Quercetin	365	2.09 ± 0.05 ^c^	1.63 ± 0.02 ^b^	1.30 ± 0.03 ^a^
Kaempferol	365	0.22 ± 0.00 ^c^	0.19 ± 0.00 ^b^	0.13 ± 0.01 ^a^
Σ Flavonols		19.34 ± 0.22 ^b^	19.19 ± 0.05 ^b^	15.04 ± 0.21 ^a^
**Anthocyanins (mg/L)**				
Delphinidin 3-O-glucoside	520	7.93 ± 0.13 ^b^	12.03 ± 0.05 ^c^	6.73 ± 0.19 ^a^
Cyanidin 3-O-glucoside	520	1.76 ± 0.09 ^b^	2.56 ± 0.11 ^c^	1.35 ± 0.03 ^a^
Petunidin 3-O-glucoside	520	14.73 ± 0.19 ^b^	21.15 ± 0.61 ^c^	12.08 ± 0.10 ^a^
Peonidin 3-O-glucoside	520	26.29 ± 0.78 ^b^	31.73 ± 1.50 ^c^	18.40 ± 0.11 ^a^
Malvidin 3-O-glucoside	520	160.27 ± 1.39 ^b^	214.95 ± 0.70 ^c^	143.08 ± 1.12 ^a^
Peonidin 3-O-(6′-acetyl)-glucoside	520	3.57 ± 0.06 ^b^	3.74 ± 0.01 ^c^	2.81 ± 0.03 ^a^
Malvidin 3-O-(6′-acetyl)-glucoside	520	14.20 ± 0.15 ^b^	18.89 ± 0.04 ^c^	13.21 ± 0.04 ^a^
Petunidin 3-(6′-*p*-coumaroyl)-glucoside	520	1.16 ± 0.01 ^b^	1.70 ± 0.04 ^c^	1.04 ± 0.00 ^a^
Malvidin 3-(6′-*p*-coumaroyl)-glucoside	520	22.74 ± 0.27 ^b^	27.98 ± 0.06 ^c^	18.92 ± 0.40 ^a^
Vitisin B Malvidin 3-O-glucoside	520	0.98 ± 0.03 ^a^	1.60 ± 0.06 ^b^	1.10 ± 0.10 ^a^
Σ Anthocyanins		253.63 ± 2.65 ^b^	336.34 ± 2.75 ^c^	218.73 ± 1.62 ^a^
**Σ Phenolic compounds (mg/L)**		352.20 ± 2.83 ^b^	412.42 ± 2.81 ^c^	295.52 ± 2.48 ^a^

C: control wine (from untreated grapevines); E: wine from grapevines treated by endotherapy; E + S: wine from grapevines treated by endotherapy and spraying; ^a^ Wavelength at which the compounds have been identified and quantified; ^b^ Coeluted compounds; ^c^ It can be glucoside or galactoside of laricitrin. All data are expressed as mean ± SD (*n* = 2). For each compound, different letters indicate significant differences according to the Tukey test (*p* < 0.05).

**Table 2:** Volatile compounds determined in Bobal wines.

	*m/z* ^a^	Odour Threshold (μg/L)	C		E		E + S	
			µg/L	OAV ^b^	µg/L	OAV ^b^	µg/L	OAV ^b^
**Acids**								
Decanoic acid	60	1000 ^c^	257.76 ± 0.33 ^a^	0.26	475.79 ± 37.60 ^b^	0.48	336.61 ± 24.90 ^a^	0.34
Hexanoic acid	60	420 ^c^	2868.58 ± 5.15 ^a^	**6.83**	4957.91 ± 285.64 ^b^	**11.80**	3094.69 ± 246.07 ^a^	**7.37**
Octanoic acid	60	500 ^c^	1348.67 ± 12.48 ^a^	**2.70**	2673.33 ± 176.03 ^b^	**5.35**	1814.57 ± 113.77 ^a^	**3.63**
Σ Acids			4475.01 ± 17.96 ^a^		8107.02 ± 499.26 ^b^		5245.87 ± 384.74 ^a^	
**Alcohols**								
Benzyl alcohol	108	200,000 ^d^	337.08 ± 8.99 ^b^	0.00	216.71 ± 14.48 ^a^	0.00	242.08 ± 4.00 ^a^	0.00
1-Hexanol	56	8000 ^e^	438.94 ± 4.68 ^ab^	0.05	476.30 ± 18.02 ^b^	0.06	411.91 ± 14.32 ^a^	0.05
2+3-Methyl-1-butanol ^h^	55	30,000 ^e^	233,096.39 ± 1509.55 ^b^	**7.77**	217,017.61 ± 3164.82 ^a^	**7.23**	211,292.30 ± 4670.82 ^a^	**7.04**
2-Phenylethanol	91	10,000 ^e^	39,573.03 ± 259.86 ^a^	**3.96**	34,542.86 ± 1799.27 ^a^	**3.45**	35,316.35 ± 1141.74 ^a^	**3.53**
Σ Alcohols			273,445.45 ± 1254.00 ^b^		252,253.48 ± 4996.58 ^a^		247,262.64 ± 3539.40 ^a^	
**Acetates**								
Ethyl acetate	43	7500 ^e^	45,936.76 ± 2241.54 ^a^	**6.12**	62,219.02 ± 711.10 ^b^	**8.30**	43,095.80 ± 748.64 ^a^	**5.75**
Hexyl acetate	43	1500 ^d^	0.91 ± 0.04 ^a^	0.00	3.13 ± 0.18 ^c^	0.00	1.40 ± 0.04 ^b^	0.00
Isoamyl acetate	43	30 ^e^	587.65 ± 9.96 ^a^	**19.59**	1499.37 ± 102.70 ^c^	**49.98**	865.31 ± 30.41 ^b^	**28.84**
Linalyl acetate	93	not found	0.10 ± 0.01 ^a^	-	0.10 ± 0.02 ^a^	-	0.08 ± 0.00 ^a^	-
2-Phenylethyl acetate	104	250 ^e^	14.69 ± 0.18 ^a^	0.06	30.29 ± 2.85 ^b^	0.12	17.71 ± 1.31 ^a^	0.07
Σ Acetates			46,540.10 ± 2251.65 ^a^		63,751.90 ± 816.85 ^b^		43,980.29 ± 716.88 ^a^	
**Ethyl esters**								
Diethyl succinate	101	200,000 ^d^	70.70 ± 0.54 ^a^	0.00	108.09 ± 9.01 ^b^	0.00	71.04 ± 4.10 ^a^	0.00
Ethyl butyrate	88	20 ^e^	99.41 ± 4.47 ^a^	**4.97**	161.79 ± 12.42 ^b^	**8.09**	106.81 ± 4.35 ^a^	**5.34**
Ethyl decanoate	43	200 ^c^	63.08 ± 9.72 ^a^	0.32	158.21 ± 21.87 ^b^	0.79	84.22 ± 6.77 ^a^	0.42
Ethyl dihydrocinnamate	104	1.6 ^c^	0.28 ± 0.01 ^a^	0.17	0.34 ± 0.03 ^a^	0.21	0.29 ± 0.02 ^a^	0.18
Ethyl hexanoate	101	14 ^c^	232.78 ± 8.78 ^a^	**16.63**	482.82 ± 46.77 ^b^	**34.49**	291.20 ± 14.98 ^a^	**20.80**
Ethyl lactate	45	154,000 ^d^	22,564.47 ± 880.50 ^b^	0.15	19,476.92 ± 484.64 ^a^	0.13	17,860.84 ± 136.98 ^a^	0.12
Ethyl octanoate	101	5 ^c^	220.22 ± 25.36 ^a^	**44.04**	499.95 ± 64.91 ^b^	**99.99**	285.03 ± 21.06 ^a^	**57.01**
Ethyl vanillate	151	990 ^f^	53.42 ± 0.86 ^a^	0.05	43.07 ± 6.01 ^a^	0.04	39.71 ± 5.46 ^a^	0.04
Σ Ethyl esters			23,304.36 ± 928.48 ^b^		20,931.19 ± 645.65 ^ab^		18,739.13 ± 193.72 ^a^	
**Terpenoids**								
Citronellol	69	100 ^d^	8.44 ± 0.10 ^b^	0.08	6.61 ± 0.24 ^a^	0.07	8.45 ± 0.36 ^b^	0.08
Farnesol	69	1000 ^g^	24.97 ± 3.10 ^a^	0.02	23.69 ± 4.35 ^a^	0.02	19.68 ± 2.30 ^a^	0.02
Geraniol	69	30 ^e^	8.49 ± 0.24 ^a^	0.28	9.56 ± 0.97 ^a^	0.32	9.21 ± 0.27 ^a^	0.31
Geranyl acetone	43	60 ^e^	0.07 ± 0.01 ^a^	0.00	0.12 ± 0.01 ^b^	0.00	0.06 ± 0.01 ^a^	0.00
*β*-Ionone	177	0.09 ^c^	0.02 ± 0.00 ^a^	0.27	0.03 ± 0.00 ^a^	0.31	0.02 ± 0.00 ^a^	0.25
Linalool	71	25 ^c^	1.38 ± 0.12 ^a^	0.06	1.95 ± 0.07 ^b^	0.08	1.83 ± 0.07 ^b^	0.07
Nerol	69	15 ^d^	3.71 ± 0.14 ^a^	0.25	3.96 ± 0.35 ^a^	0.26	4.47 ± 0.04 ^a^	0.30
Nerolidol	69	15 ^d^	1.39 ± 0.18 ^a^	0.09	2.05 ± 0.27 ^a^	0.14	1.88 ± 0.15 ^a^	0.13
Σ Terpenoids			48.47 ± 2.93 ^a^		47.98 ± 6.27 ^a^		45.61 ± 3.20 ^a^	
**Volatile phenols**								
Eugenol	164	6 ^c^	1.11 ± 0.02 ^a^	0.18	1.11 ± 0.17 ^a^	0.19	1.22 ± 0.06 ^a^	0.20
Guaiacol	109	9.5 ^c^	69.53 ± 12.84 ^a^	**7.32**	89.01 ± 25.10 ^a^	**9.37**	71.75 ± 1.15 ^a^	**7.55**
Σ Volatile phenols			70.64 ± 12.86 ^a^		90.12 ± 25.27 ^a^		72.97 ± 1.09 ^a^	

C: control wine (from untreated grapevines); E: wine from grapevines treated by endotherapy; E + S: wine from grapevines treated by endotherapy and spraying. ^a^ *m*/*z* with which the compounds have been identified and quantified. ^b^ OAV: Odour activity value, shown in bold for compounds with concentrations above their odour threshold (the superscript letters indicate the references for the odour threshold of each compound: ^c^ Ferreira et al. (2000) [52]; ^d^ Etiévant (1991) [53]; ^e^ Guth (1997) [54]; ^f^ López et al. (2002) [55]; ^g^ Franco et al. (2004) [56]. ^h^ Coeluted compounds. All data are expressed as mean ± SD (*n* = 2). For each compound, different letters indicate significant differences according to the Tukey test (*p* < 0.05).

## Data Availability

The data presented in this study are available on request from the corresponding author.

## Sample Availability

Samples of the compounds are not available from the authors.
